# Analysis of Changes in Scalp Skin Thickness According to Age and Sex Based on Ultrasonography and Histometry

**DOI:** 10.1055/a-2646-8750

**Published:** 2025-09-01

**Authors:** Kun Yong Sung, Seung Ho Lee, Suk Joon Oh, Chanho Jeong, Jong Dae Kim, Jeong Tae Kim

**Affiliations:** 1 Department of Plastic and Reconstructive Surgery, Kangwon National University Hospital, Kangwon, Republic of Korea; 2 Department of Burn Reconstructive Surgery, Daejeon Hwa Hospital, Daejeon, Republic of Korea; 3 Department of Plastic and Reconstructive Surgery, Bestian Seoul Hospital, Seoul, Republic of Korea

**Keywords:** histometry, scalp skin grafting, scalp skin thickness, ultrasonography

## Abstract

**Background:**

This study investigates scalp skin thickness using ultrasonography and histometry, exploring its relationship with age and sex.

**Methods:**

The study data were obtained from split-thickness skin grafting using hairy scalp skin as the donor site in 102 Korean patients. The skin thickness of the donor scalps was measured using preoperative ultrasound in all 102 patients, and the histometric thickness of a 3-mm punch biopsy near the donor site was measured in 61 patients postoperatively. The scalp skin thickness was statistically analyzed according to age and sex.

**Results:**

The mean ultrasonographic thickness was 1.71 ± 0.41 mm, while the mean histometric thickness was 1.93 ± 0.47 mm. The difference was statistically significant ( *p* < 0.001). Regression analysis showed a significant relationship between ultrasonographic scalp skin thickness and age, but not sex. Thickness increased up to 20 years, with no significant change beyond that.

**Conclusion:**

Scalp skin thickness correlates with age, but not sex. Preoperative ultrasonography is crucial for assessing scalp skin thickness, especially for younger patients under 21 years, to optimize scalp grafting outcomes.

## Introduction

The human scalp skin thickness varies with age and sex, with previous studies showing differences in both histometric and ultrasonographic measurements across individuals. 1 The non-invasive ultrasound technique provides a precise method for measuring skin thickness without the risks of radiation. 2

In previous research, the skin thickness on the flexor aspect of the right mid-forearm showed a linear increase in younger individuals and a decrease in older groups (21 years and above). 3

This study investigates the relationship between scalp skin thickness, age, and sex using both high-frequency ultrasonography and histometry, aiming to provide critical insights into precise surgical planning in scalp skin grafting.

The hair follicle consists of the proximal dermal follicle bulge (permanent) and the distal hair shaft (cycling), with their relative positions depending on the growth phase of the hair cycle. In the telogen phase (resting phase), the hair bulb is located in the reticular dermis, while in the anagen phase (active growth), it is found near the subcutaneous tissue. During the catagen phase (transition), the hair bulb is in an intermediate position. 4 5

Furthermore, in the telogen phase, the germinal unit and the upper part of the follicular stelae are positioned beneath the follicular bulge, residing within the reticular dermis of the human scalp. 6 In a study by de Viragh and Meuli, 4 the development of various follicular compartments, including the infundibulum, bulge/isthmus, Adamson's fringe, B-fringe, and the matrix, was assessed by measuring their depths in parietal scalp biopsy specimens from 100 patients ranging from 2 weeks to 21 years of age. 7

If the thick split-thickness skin graft includes the dermal papilla cells and telogen germinal units of the hair follicle in the lower part of the hair bulge, the grafted skin can develop hair transplants. Therefore, the thickness of the scalp skin must be determined before surgery because the risk of hair transfer can be avoided if the thick split-thickness skin graft is harvested at the level of the infundibulum of the scalp hair.

In this context, our study aims to comprehensively investigate the relationship between scalp skin thickness, age, and sex using high-frequency ultrasonography and histometric analysis. By establishing reliable baseline data and identifying age-specific variations, we aim to enhance the precision and safety of scalp-related surgical procedures, including split-thickness skin grafting.

## Methods

This study was approved by the Institutional Review Board (IRB approval no. BMC 2024-05-007) and was conducted in accordance with the principles of the Declaration of Helsinki. Written informed consent was obtained from all patients for participation in the study and the use of their data in academic publications.

The study data were obtained from thick split-thickness skin grafting using scalp skin as the donor site in 102 Korean patients (70 males and 32 females) with a mean age of 33.3 ± 23.8 years (range: 0.8–78 years). The indications for recipient site grafting included acute burn wounds, postburn scar contractures, traumatic wounds, flap or graft loss wounds, posttraumatic graft scars, and irradiated skin lesions. Among these, postburn scar contractures were the most common, accounting for 60 of 102 cases.

The hairy scalp skin thickness at the temporoparietal region was measured using high-frequency ultrasonography (Esaote MyLab One, Genoa, Italy; 18 MHz) in all 102 patients prior to surgery. To minimize the potential measurement error due to probe pressure, an Aquaflex Ultrasonic Gel Pad (2 × 9 cm, Parker Labs) was used during ultrasonographic measurements.

The histometric thickness of a 3-mm punch biopsy adjacent to the donor site was measured in 61 patients after surgery. Separate explanations were provided for both the ultrasonographic and histologic measurement procedures, ensuring that patients were fully informed about each method and its implications. Of the 102 patients initially considered, 41 declined to undergo the biopsy procedure and were therefore excluded from the histometric analysis. All patients who underwent the biopsy voluntarily consented to the procedure after the risks and purpose had been thoroughly explained.

### Statistical Analysis

All statistical analyses were performed using R Statistical Software (version 4.0.3, R Foundation for Statistical Computing, Vienna, Austria). Continuous variables were summarized using both mean ± standard deviation (SD) and median with interquartile range (IQR), in order to provide a comprehensive description of data distribution and assess potential skewness.

The normality of continuous variables was assessed using the Shapiro–Wilk test. As the assumption of normality was not satisfied in some groups, and considering the small sample sizes in certain comparisons, non-parametric tests were employed instead of parametric tests for the comparison of representative values. For modeling the relationship between variables, simple linear regression analysis was performed. As the assumption of normality is not required for the variables themselves but for the residuals, residual diagnostics were conducted to validate the regression model, including assessments of residual normality (via Q–Q plots and the Shapiro–Wilk test) and homoscedasticity (via residual plots and the Breusch–Pagan test).

The Wilcoxon signed-rank test was used to compare ultrasonographic and histometric scalp thickness measured in the same subjects. To evaluate their association, Spearman's rank correlation and simple linear regression analysis were conducted.

The Wilcoxon rank-sum test was used to compare scalp skin thickness between male and female subjects within each age subgroup (≤20 and >20 years). The 95% confidence intervals for the median differences were estimated using non-parametric bootstrap resampling.

Multiple linear regression analysis was conducted to investigate the association between ultrasonographic scalp thickness and potential predictors, including age and sex. Subgroup analyses were performed for participants younger than 21 years and those aged 21 years and older. For each regression model, the regression coefficient, standard error (SE), *R* ^2^ value, *t* -value, and *p* -value were reported.

All statistical tests were two-sided, and a *p* -value less than 0.05 was considered statistically significant.

## Results

### Comparison between Ultrasonographic and Histometric Measurements

In 61 cases where both methods were utilized, the mean ultrasonographic thickness of normal scalp skin was 1.71 ± 0.41 mm (mean ± SD), with a median of 1.80 mm (IQR: 1.40–2.00 mm). The corresponding histometric thickness was 1.93 ± 0.47 mm (mean ± SD), with a median of 1.90 mm (IQR: 1.56–2.25 mm). Additionally, the Wilcoxon signed-rank test showed that the median paired difference in thickness between the two methods was 0.20 mm (IQR: 0.00–0.42 mm), which was statistically significant ( *p* < 0.001). A strong positive correlation was observed between ultrasonographic and histometric measurements, with a Spearman's rank correlation coefficient of 0.71 ( *p* < 0.001; Table 1 ).

**Table 1:** Comparison of ultrasonographic and histometric scalp skin thickness (mm)

	*N*	Mean (SD)	Median (IQR)	Median paired differences (IQR)	*p* -Value for Wilcoxon signed-rank test	ρ
Ultrasound (in vivo)	61	1.71 (0.41)	1.80 (1.40–2.00)	0.20 (0.00–0.42)	<0.001	0.71
Histometric (in vitro)	61	1.93 (0.47)	1.90 (1.56–2.25)			

Abbreviations: IQR, interquartile range; *N* , number; SD, standard deviation; ρ, Spearman's rank correlation coefficient.

The simple linear regression model for the relationship between ultrasonographic and histometric thickness was

Histologic thickness (mm) = 0.47 + 0.85 × ultrasonographic thickness (mm) ( *R* ^2^ = 0.53, SE = 0.10, *t* = 8.17, *p* < 0.001)

**Fig. 1:**
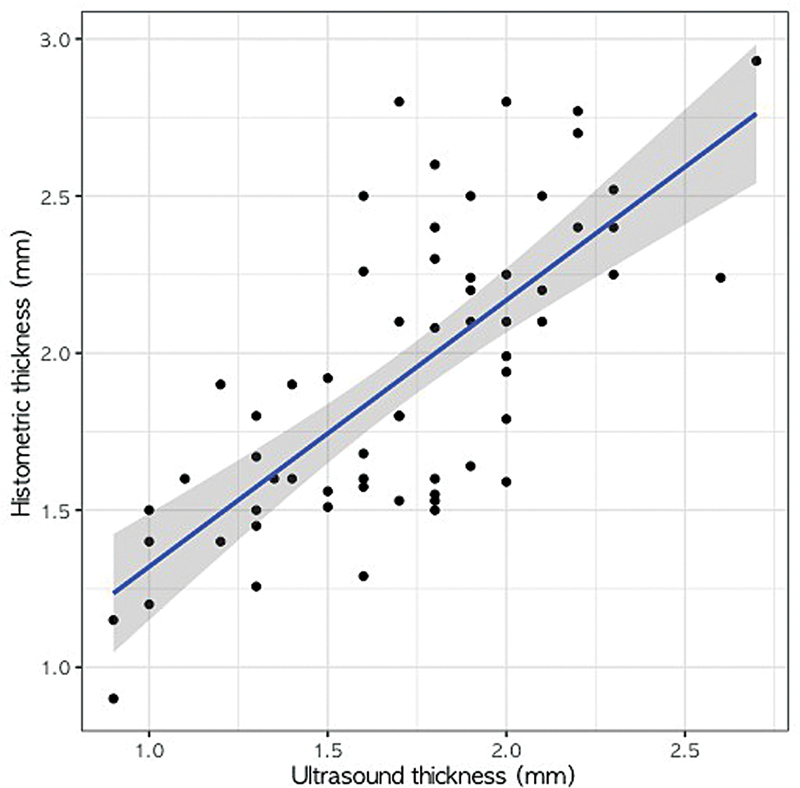
Relationship between the histometric thickness and ultrasound thickness of normal scalp skin.

This indicates a statistically significant positive correlation between the two methods of measuring scalp skin thickness ( Fig. 1 ). Residual diagnostics confirmed that the assumptions of normality and homoscedasticity were satisfied.

### Comparison of Scalp Skin Thickness by Age and Sex

In the group below 21 years of age, the mean ultrasonographic scalp thickness was 1.36 ± 0.33 mm (mean ± SD), with a median of 1.33 mm (IQR: 1.10–1.58 mm) in males, and 1.36 ± 0.30 mm (mean ± SD), with a median of 1.30 mm (IQR: 1.20–1.63 mm) in females.

In the group of patients aged 21 years and above, the mean ultrasonographic scalp thickness was 1.99 ± 0.31 mm (mean ± SD), with a median of 1.95 mm (IQR: 1.78–2.11 mm) in males, and 1.90 ± 0.28 mm (mean ± SD), with a median of 1.90 mm (IQR: 1.80–1.98 mm) in females.

The Wilcoxon rank-sum test showed that the median difference by sex was not statistically significant in either age group ( *p* = 0.989 for under 20 years, *p* = 0.262 for over 20 years; Table 2 ). This indicates that sex does not have a substantial effect on scalp skin thickness in either age group.

**Table 2:** Scalp skin thickness according to sex and age group (mm)

		*N*	Mean (SD), mm	Median, mm (IQR)	Median differences (95% CI)	*p* -Value for Wilcoxon rank-sum test
Under 20 years	Female	12	1.36 (0.30)	1.3 (1.20–1.63)	0.03 (−0.3994, 0.3000)	0.989
	Male	30	1.36 (0.33)	1.33 (1.10–1.58)		
Over 20 years	Female	20	1.90 (0.28)	1.90 (1.80–1.98)	0.05 (−0.05, 0.20)	0.262
	Male	40	1.99 (0.31)	1.95 (1.78–2.11)		

Abbreviations: CI, confidence interval; IQR, interquartile range; *N* , number; SD, standard deviation.

### Regression Analysis for Age and Ultrasonographic Scalp Skin Thickness

**Fig. 2:**
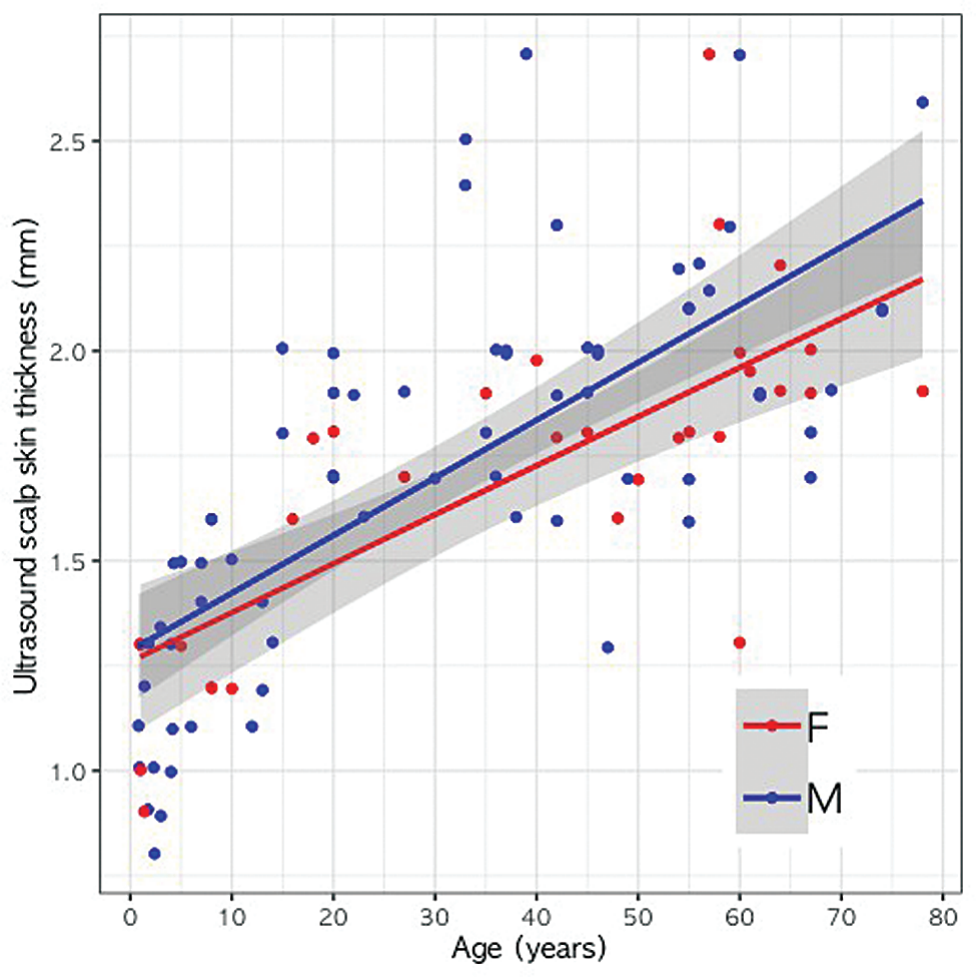
Relationship between ultrasonographic scalp skin thickness and age within each sex. In the figure, red color represents females, while blue represents males. Solid lines mean the simple linear regression line for each sex.

The results of a multiple linear regression analysis indicated that ultrasonographic scalp skin thickness showed a statistically significant relationship with age (SE = 0.001, *t* -value = 10.373, *p* < 0.001), but not with sex (SE = 0.064, *t* -value = 1.542, *p* = 0.126; Fig. 2 ).

In the group below 21 years of age (42 cases), the regression equation relating age and ultrasonographic scalp skin thickness was as follows:

Scalp thickness (mm) = 1.042 + 0.037 × age (years) ( *R* ^2^ = 0.610, SE = 0.005, *t* -value = 7.914, *p* < 0.001)

In the group of patients aged 21 years and above (60 cases), the regression equation was as follows:

Scalp thickness (mm) = 1.779 + 0.004 × age (years) ( *R* ^2^ = 0.026, SE = 0.003, *t* -value = 1.253, *p* = 0.215)

**Fig. 3:**
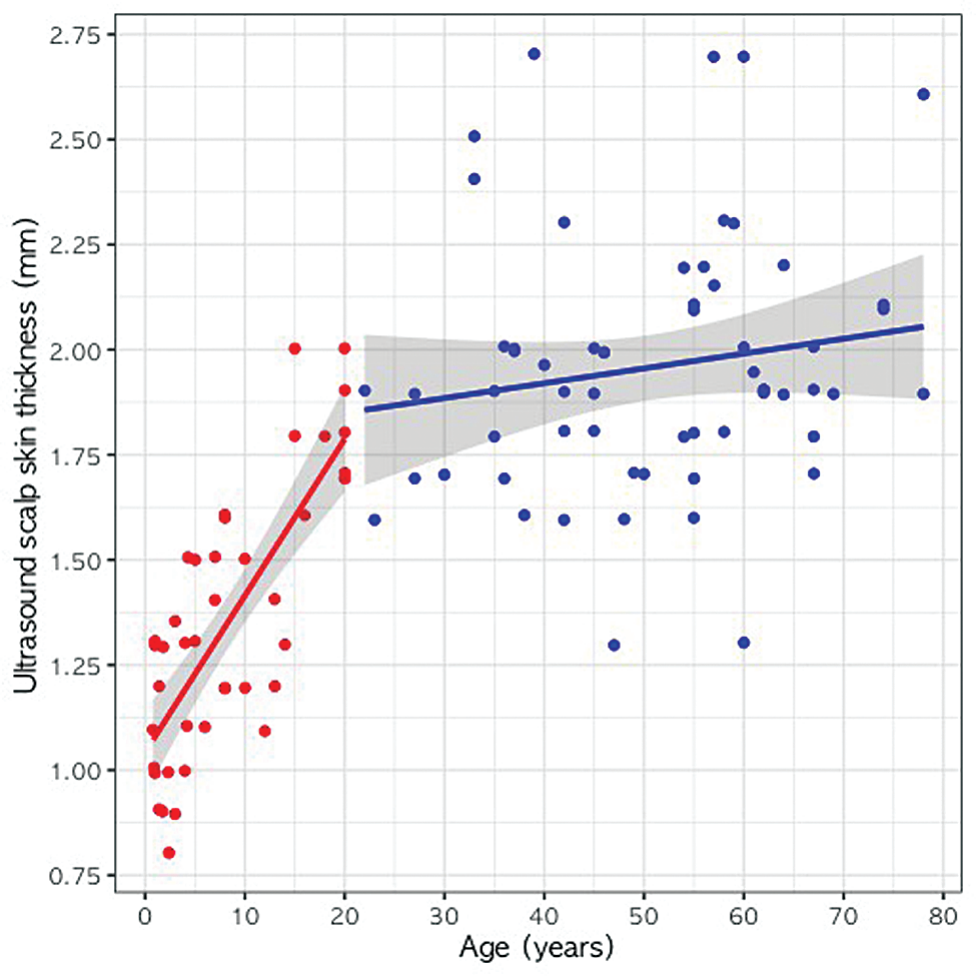
Relationship between ultrasound scalp skin thickness and age within each age group. Red color means under 20 years old, and blue color means over 20 years old. Solid lines mean the simple linear regression line for each age group, and the gray area means the 95% confidence interval.

This finding suggests that scalp skin thickness gradually increases with age up to approximately 20 years, whereas no substantial association between age and scalp skin thickness is observed thereafter ( Fig. 3 ).

### Regression Analysis for Age and Histometric Scalp Skin Thickness

Subgroup analyses were conducted for histometric scalp skin thickness. In the group below 21 years of age (24 cases), the regression equation relating age and histometric scalp skin thickness was as follows:

Scalp thickness (mm) = 1.303 + 0.040 × age (years) ( *R* ^2^ = 0.546, SE = 0.008, *t* -value = 5.141, *p* < 0.001)

In the group of patients aged 21 years and above (37 cases), the regression equation was as follows:

Scalp thickness (mm) = 2.158 − 0.0005 × age (years) ( *R* ^2^ = 0.0003, SE = 0.005, *t* -value = 0.095, *p* = 0.925)

**Fig. 4:**
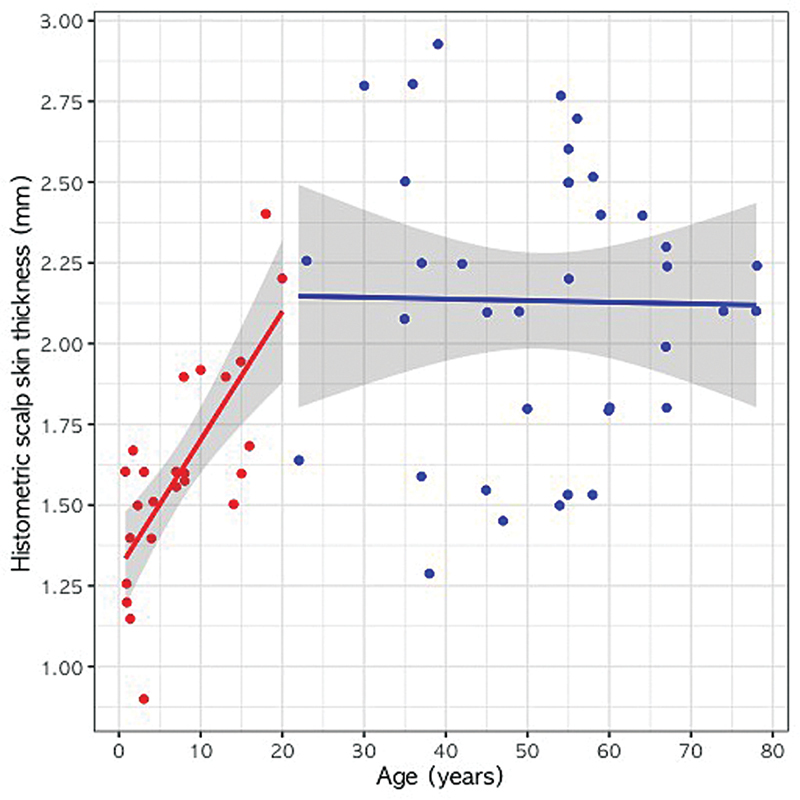
Relationship between histometric scalp skin thickness and age within each age group. Red color means under 20 years old, and blue color means over 20 years old. Solid lines mean the simple linear regression line for each age group, and the gray area means the 95% confidence interval.

Similar results were obtained for histometric scalp skin thickness, showing a pattern consistent with that observed for ultrasonographic measurements ( Fig. 4 ). This indicates that histometric thickness increases with age up to 20 years, but no significant relationship was observed beyond that.

## Discussion

Our study aimed to evaluate scalp skin thickness using ultrasonography and histometry and investigate the relationship between age, sex, and scalp skin thickness. The main findings of this study can be summarized as follows. The mean ultrasonographic thickness of normal scalp skin was 1.71 mm, and the mean histometric thickness was 1.93 mm, with a statistically significant difference of 0.212 mm ( *p* < 0.001). A strong positive correlation ( *r* = 0.71, *p* < 0.001) was found between ultrasonographic and histometric measurements. Furthermore, simple linear regression analysis showed a significant linear relationship between the two methods, with the regression equation:

Histologic thickness (mm) = 0.47 + 0.85 × ultrasonographic thickness (mm) ( *R* ^2^ = 0.53, *p* < 0.001).

This suggests that ultrasonographic measurements can reliably predict histometric thickness, supporting the use of ultrasonography as a non-invasive tool for accurate scalp skin thickness measurement in clinical practice. Age showed a statistically significant relationship with scalp skin thickness up to 20 years ( *p* < 0.001), while sex did not ( *p* = 0.126).

High-frequency ultrasonography is a valuable tool for understanding the normal structure of human skin and for comparing histometric and ultrasonographic findings. 8 However, recent studies utilizing high-frequency ultrasonography to measure scalp skin thickness are scarce. Our study provides valuable insights into scalp skin thickness, offering essential baseline data for scalp skin grafting procedures. In our study, Spearman's rank correlation of 0.71 further confirms the consistency of the findings with prior research demonstrating a strong correlation between ultrasonographic and histometric measurements of skin thickness. 9 This highlights the reliability of ultrasonography as a non-invasive and accurate method for measuring scalp skin thickness in clinical practice. To address this, we employed a complementary approach using histological thickness data from 3-mm punch biopsy samples to validate the accuracy and reliability of the ultrasound-derived thickness measurements. This dual-modality assessment was critical to ensure the robustness of our findings and to determine the degree of concordance between ultrasonographic and histologic scalp thickness measurement, and suggested that future research in this field can confidently rely on non-invasive ultrasonographic assessment alone.

The comparison between ultrasonographic and histometric measurements in this study revealed a statistically significant difference, with histometric measurements of scalp thickness being 12.4% higher than those obtained via ultrasonography. The difference in thickness between the two methods was statistically significant, as confirmed by the paired *t* -test ( *p* < 0.001) and the Wilcoxon signed-rank test ( *p* < 0.001). This finding is consistent with prior studies on forearms, which also reported higher histometric measurements than ultrasonographic ones, probably resulting from the release of internal tension in the dermis following tissue removal. 3

In our study, the results of a multiple linear regression analysis indicated that ultrasonographic scalp skin thickness showed a statistically significant relationship with age, but not with sex. Scalp skin thickness increases progressively until the age of 20, after which the association between age and thickness diminishes significantly. This observation is consistent with findings from studies on male pattern baldness and other studies of skin aging. 1 4 These findings highlight the need for age-specific grafting strategies in younger patients, as thinner grafts may be required to avoid complications such as hair transfer and alopecia. 10 11

The clinical significance of this study lies in demonstrating the importance of preoperative high-frequency ultrasonography for assessing scalp skin thickness, particularly in patients under 21 years of age. Ultrasonography provides a non-invasive, real-time method that allows for precise preoperative evaluation of graft thickness, thereby minimizing potential complications. In our study, the strong correlation (Spearman's ρ = 0.71) between ultrasonographic and histometric thickness measurements further supports the reliability of ultrasonography as an accurate alternative to histologic data. Additionally, age was found to significantly influence scalp skin thickness up to 20 years, underscoring the necessity for age-specific grafting strategies in younger patients. Future research should focus on determining the optimal graft depth while considering factors such as infundibulum depth, scalp thickness, the potential influence of comorbidities, and the risk of hair transfer during grafting procedures.

### Conclusion

This study confirmed a strong correlation between ultrasonographic and histometric measurements of scalp skin thickness, highlighting ultrasonography as a reliable, non-invasive tool for preoperative assessment. Age was found to significantly influence scalp skin thickness up to 20 years, highlighting the importance of age-specific grafting strategies in younger patients. In adults, the correlation was negligible ( *R* ^2^ = 0.026). These findings provide a valuable foundation for future research aimed at refining scalp-related surgical approaches.

### Limitations

One of the limitations of this study is the relatively small sample size in certain subgroups, particularly in females under 20 years of age. This may reduce the statistical power and limit the generalizability of the findings to broader populations. Although non-parametric tests were used to account for small sample sizes and non-normal distributions in these subgroups, the limited number of observations may still affect the robustness and external validity of the subgroup analyses.

Another limitation is the potential for minor residual compression effects during ultrasonographic measurements, even though we employed a gel pad to minimize probe pressure. This might have contributed to measurement variability.

Additionally, the correlation between age and scalp thickness was negligible in adults ( *R* ^2^ = 0.026), suggesting that age may have limited utility as a predictor of scalp thickness in this population.
